# The same and not the same: heterogeneous functional activation of prostate tumor cells by TLR ligation

**DOI:** 10.1186/1475-2867-14-54

**Published:** 2014-06-19

**Authors:** Simin Rezania, Noor Amirmozaffari, Nesa Rashidi, Ebrahim Mirzadegan, Saeed Zarei, Jamileh Ghasemi, Omid Zarei, Leila Katouzian, Amir-Hassan Zarnani

**Affiliations:** 1 Department of Microbiology, School of Medicine, Iran University of Medical Sciences, Tehran, Iran; 2 Biophysics Institute, Medical University of Graz, Graz, Austria; 3 Reproductive Biotechnology Research Center, Avicenna Research Institute, ACECR, Tehran, Iran; 4 Reproductive Immunology Research Center, Avicenna Research Institute, ACECR, Tehran, Iran; 5 Monoclonal Antibody Research Center, Avicenna Research Institute, ACECR, Tehran, Iran; 6 Nanobiotechnology Research Center, Avicenna Research Institute, ACECR, Tehran, Iran; 7 Department of Pharmaceutical Biotechnology, Faculty of Pharmacy, Tabriz University of Medical Sciences, Tabriz, Iran; 8 Immunology Research Center, Iran University of Medical Sciences, Tehran, Iran

**Keywords:** Toll like receptor, Prostate cancer, Proliferation, Pro-inflammatory cytokine, Invasion, Adhesion, Inflammation

## Abstract

**Background:**

Many types of tumors are organized in a hierarchy of heterogeneous cell populations with different molecular signature. Such heterogeneity may be associated with different responsiveness to microenvironment stimuli. In the present study, the effects of lipopolysaccharide (LPS) and lipoteichoic acid (LTA), as well-known mediators of inflammation, on cancerous behavior of three prostate tumor cells, LNCaP, PC3 and DU145, were investigated.

**Methods:**

Expression of TLR1-10, CD14 and MyD88 transcripts was investigated by RT-PCR. Protein expression of TLR2 and 4 was scrutinized by flow cytometry, immunofluorescent staining and Western blotting. Experiments were set up to assess the effects of LPS and LTA at different concentrations and times on cell proliferation, extracellular matrix invasion, adhesion and cytokine production.

**Results:**

We showed that prostate cancer cell lines differentially express TLR1-10, MyD88 and CD14 transcripts. DU145 failed to express TLR4 gene. Positively-identified TLR2 protein in all prostate cancer cells and TLR4 protein in PC3 and LNCaP by Western blotting was not accompanied by cell surface expression, as judged by flow cytometry. Immunofluorescent staining clearly demonstrated predominantly perinuclear localization of TLR2 and TLR4. LTA activation of all prostate cancer cells significantly increased cell proliferation. Regardless of lacking TLR4, DU145 cells proliferated in response to LPS treatment. While LPS caused increased invasiveness of LNCaP, invasive capacity of PC3 was significantly reduced after LPS or LTA stimulation. Stimulation of all prostate tumor cells with LTA was associated with increased cell adhesion and IL-8 production. IL-6 production, however, was differentially regulated by LPS stimulation in prostate tumor cells.

**Conclusion:**

The data shows that cancer cells originated from the same histologically origin exhibit heterogeneous response to the same TLR ligand. Therefore, a thorough and comprehensive judgment on how and to what extent a particular cancer is affected by TLR agonist could not be inferred by studying an individual cell line.

## Introduction

Immune recognition of microorganisms is carried out by a set of receptors collectively referred to as pattern recognition receptors (PRR). These receptors are expressed by wide variety of immune cells and, as an essential part of innate immunity, are involved in immediate and direct recognition of molecular determinants specific to certain classes of pathogens [1]. TLRs are more recent class of PRR which comprise a family of at least ten molecules expressed in wide range of organisms from *Drosophila* to higher mammals [2-4]. Each individual TLR is believed to recognize specific classes of microbial determinants. TLRs 2, 3, 4, 5, 7 and 9 sense bacterial lipoproteins, double-stranded RNA/poly (I:C), lipopolysaccharides, flagellin, single stranded RNA and CPG-containing DNA, respectively [5-14]. Most TLRs including TLR2 and 4 signal through a common adaptor protein, myeloid differentiation primary response gene 88 (MyD88). Following TLR ligation, recruitment of MyD88 takes place which in turn associates with the intracellular domain of the TLR [15-18] leading to subsequent downstream activation of the nuclear factor, NF-kB, signaling pathway. The latter is responsible for the initiation of pro-inflammatory responses characterized by the production of a vast array of chemokines and cytokines and in some cell populations by cell proliferation, as well [19]. Although most of the studies on TLRs published so far have focused on their expression and function in immune cells, there are accumulating set of evidence indicating that other cell types including epithelial cells and cancer cells of different origin also express TLRs [20,21].

It is widely accepted that chronic inflammation is among the main triggers of tumorigenesis [22] and in this regard cancer cells may benefit from inflammatory process through expression of TLRs leading to further propagation and development of chemoresistance. There are plenty of reports providing compelling evidence supporting the role of inflammatory process induced by bacterial and viral components in carcinogenesis or alteration of invasive behavior of previously-established tumors [23-25].

Attempting to explore the TLR biology in cancer, several research projects have been carried out with cell lines affiliated to the solid tumors of different origin including colon, breast, prostate, melanoma, lung, larynx, neuroblastoma, ovary and cervix, to list a few [26-33]. In most settings, however, the expression pattern has been surveyed at the gene level only and data on functional expression of TLRs on cancer cells is rather elusive with contradictory results. According to some reports, TLR engagement leads to production of pro-inflammatory factors such as IL-12, IL-6 and nitric oxide by tumor cells and results in their resistance to cytotoxcicity and apoptosis, increased invasiveness, chemoresistance and tumor growth [24,28,34-39]. In contrast, some tumors are unresponsive to TLR ligands regardless of possessing all the downstream molecules required for TLR signaling [27,40].

In developed countries, prostate cancer is the most common cancer in men, and it ranks third overall in terms of mortality [41]. A great body of evidence supports the hypothesis that environmental factors such as chronic inflammation and infection are important for development of prostate cancer [42]. It has been shown that LNCaP prostate cancer cells, when exposed to the conditioned media of LPS-activated THP-1 macrophage cells, produce pro-inflammatory cytokines and upregulate markers associated with cell immune evasion and tumor progression [43]. Nonetheless, there is a great debate on pro- and anti-tumoral activity of different TLRs in prostate cancer [44]. Based on the fact that many types of tumors are organized in a hierarchy of heterogeneous cell populations, we hypothesized that such skepticism may stem from different behavior of prostate cancer cells with different molecular signatures. Therefore we examined and compared expression of TLR1-10, MyD88 and CD14 and functional responsiveness to TLR-2 and 4 ligands in well-established prostate cancer cell lines.

## Results

### Expression of TLRs, CD14 and MyD88 transcripts in prostate cancer cell lines

**Figure 1:**
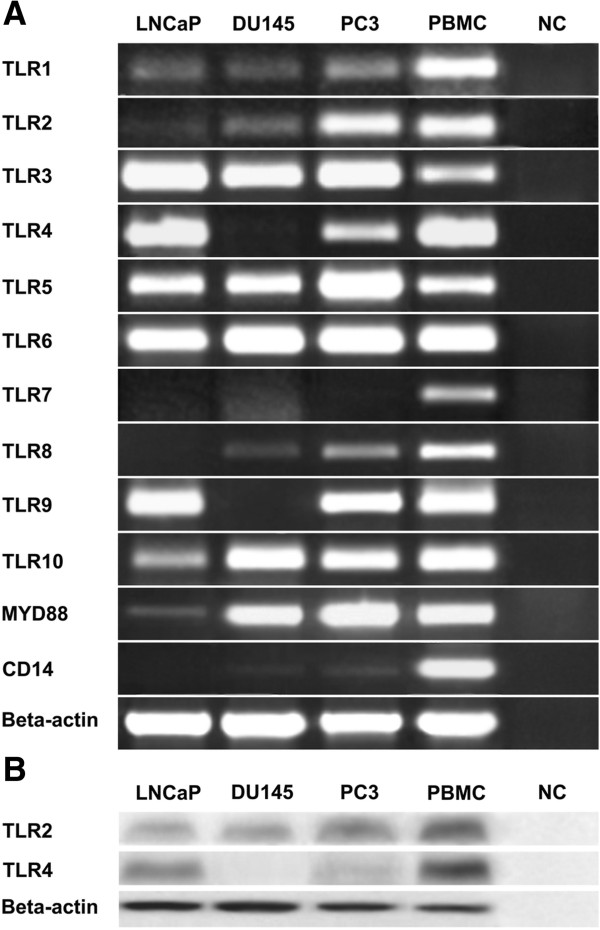
**Expression of TLRs in human prostate cancer cell lines. A**: RT-PCR showing expression of TLR1-10, MyD88 and CD14 transcripts. RT-PCR was performed according to the protocol described in Materials and methods. PBMC served as positive control. Amplification of Beta-actin in the same samples was used as loading control. No amplification controls (NC)(no reverse transcriptase added) were included as negative controls in all PCR programs. **B**: Western blot analysis of TLR2 and TLR4 expression. Positive sample and loading controls were included as RT-PCR experiments. Lanes in which primary antibodies were substituted by non-immune sera served as negative controls (NC). Similar results were observed in three independent experiments.

Expression of TLR1-10, MyD88 and CD14 in prostate cancer cells, LNCaP, PC3 and DU145, was studied by RT-PCR. Results are shown in Figure 1A. PC3 and DU145 expressed CD14 above detection limit, while LNCaP failed to express this marker. TLR1-10 and MyD88 were differentially expressed by these cell lines with different densities. Of the TLR genes examined, LNCaP failed to express detectable levels of TLR7 and 8 and exhibited very low level expression of TLR2, while DU145 expressed all TLRs except TLR4, TLR7 and 9. Except TLR7, PC3 expressed all TLR transcripts. All cell lines expressed MyD88.

### Expression of TLR2 and TLR4 proteins in prostate cancer cell lines

**Figure 2:**
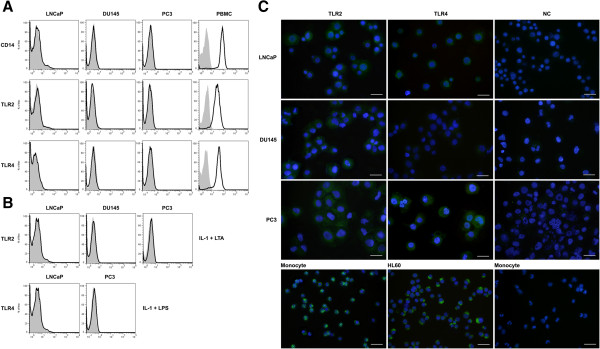
**Flow cytometric analysis of TLR2 and TLR4 expression in human prostate cancer cell lines. A**: Basal levels of TLR2, TLR4 and CD14 expression was assessed using specific antibodies (white areas) or isotype controls (gray areas). Monocyte gate of PBMC served as positive control. **B**: Cells were stimulated with a combination of LPS + IL-1 or LTA + IL-1 and expression of TLR2 and TLR4 was evaluated as above. The diagrams are representative of at least three independent experiments. **C**: Immunofluorescent localization of TLR2 and TL4 in prostate cancer cell lines. MACS-purified human monocytes and HL-60 cell line served as positive control for TLR2 and TLR4 expression, respectively. Scale bare: 50 μm.

In order to investigate TLR2 and TLR4 protein expression and their subcellular localizations, three protein readout systems were employed. In Western blot analysis, specific bands of about 100 and 95 KDs were detected in PBMC (as positive control) for TLR2 and TLR4, respectively. In line with PCR results, cell lines with positive gene expression also expressed corresponding proteins (Figure 1B). DU145 failed to express TLR4 protein. Surface expression of TLR2 and TLR4 proteins in prostate cell lines was examined by flow cytometric analysis. Monocyte gate of PBMC served as positive control. Our results clearly showed that, none of the cell lines expressed TLR2 or TLR4 on their surface. Indeed, no detectable levels of surface CD14 was found in prostate cancer lines. Monocytes as positive control expressed high levels of TLR2, TLR4 and CD14 on their surface (Figure 2A). To test whether surface expression of TLR2 and 4 is affected by LPS, LTA or IL-1, they were pretreated with a mixture of LPS + IL-1 or LTA + IL-1 depending on expression of TLR2 and TLR4 transcripts. The results clearly showed that such treatments could not up regulate surface expression of TLR2 and 4 in prostate cancer cell lines (Figure 2B). Based on the results of Western blot and flow cytometric analyses, we were then about to localize TLR2 and TLR4 expression in prostate cancer cells. In immunofluorescent staining, TLR2 and TLR4 were mainly localized to the peri-nuclear region of the cells (Figure 2C).

### Effects of TLR ligands on prostate cancer cell proliferation

**Figure 3:**
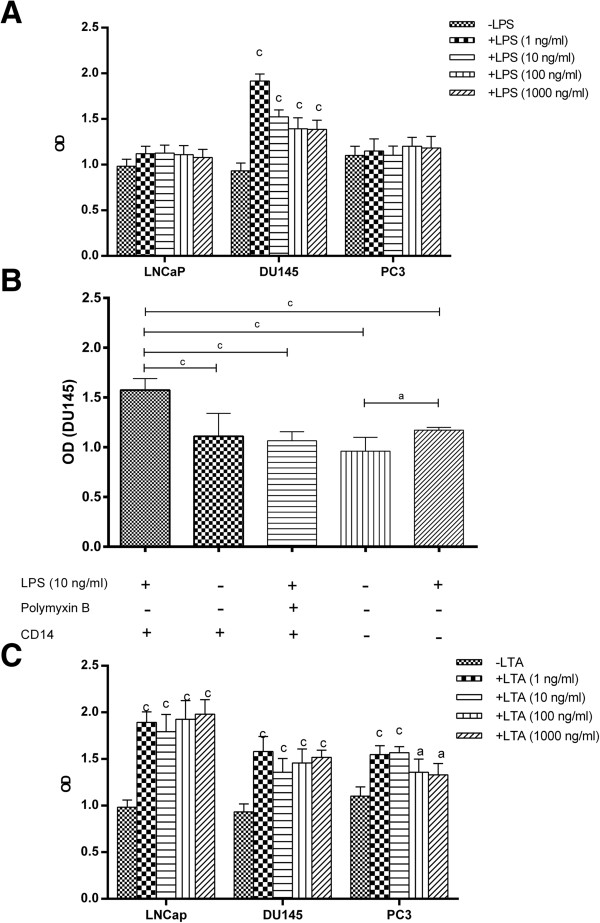
**Effects of TLR activation on proliferation of human prostate cancer cell lines.** The effects of LPS **(A)** and LTA **(C)** on proliferation of prostate cancer cell lines were examined. Cells were treated with a wide concentration range of LPS or LTA and their proliferation was measured by XTT assay in reference to vehicle-treated cells. To rule out the possibility of contamination of LPS with other TLR ligands, LPS was neutralized by Polymyxin B and the level proliferation in DU145 cells was assessed either in the presence or absence of soluble CD14 **(B)**. Each bar represents the mean ± SD of three independent experiments and each test was done in triplicate. In each cell line, the data were compared to vehicle-treated (control) cells. c: p < 0.001, a: p < 0.05.

Uncontrolled cell proliferation is without any doubt a hallmark of cancerous cells and in this regard microenvironment in which the cells reside has fundamental effect on this behavior. TLR expression by these cells may influence their proliferation rate through receptor-ligand engagement. To this end, the effect of TLR4 and TLR2 ligands, LPS and LTA, on proliferation of prostate cancer cell lines was examined. To do this, cells were treated by a broad concentration range of aforesaid ligands and their proliferation was measured by XTT assay in reference to unstimulated cells. Although, DU145 cells failed to express TLR4 transcript, they also stimulated by LPS to see whether presence of specific ligands are necessary to induce functional proliferative response. The result showed that different prostate cell lines had completely different proliferative response to TLR2 and 4 ligands (Figure 3). Although DU145 did not express cognate receptor for LPS, their proliferation capacity was significantly increased in response to different concentrations of LPS (p < 0.001) (Figure 3A). Although we used highly pure LPS, to rule out the possibility of contamination of LPS with other TLRs ligands we performed cell proliferation assay in DU145 cells using Polymyxin B to neutralize LPS. The results of such experiment clearly showed that LPS neutralization lowered proliferative capacity of DU145 cells to the baseline level (p < 0.001). Also, omission of exogenous CD14 significantly abolished LPS-induced proliferation of this cell line (p < 0.05) (Figure 3B).LPS treatment did not alter proliferation of LNCaP or PC3 regardless of TLR4 expression (Figure 3A). LTA treatment at all concentrations significantly increased proliferation of LNCaP, DU145 and PC3 cells (p < 0.05 or p < 0.001 depending on cell type and LTA concentration) (Figure 3C). Interestingly, as kinetic study demonstrated, responder cells also showed considerably varying response to the different concentrations of LPS or LTA. In the case of DU145, low concentrations of LPS were more effective in triggering proliferative response compared to very high concentration (1000 ng/ml). The same pattern was also seen when PC3 cell line was treated with LTA. Considering different basal level proliferation in cell lines tested, stimulation index (SI) was calculated and compared. In response to LTA treatment, DU145 had the highest SI in all concentrations examined (data not shown).

### Effects of TLR2 and TL4 ligation on cytokine production by prostate cancer cells

**Figure 4:**
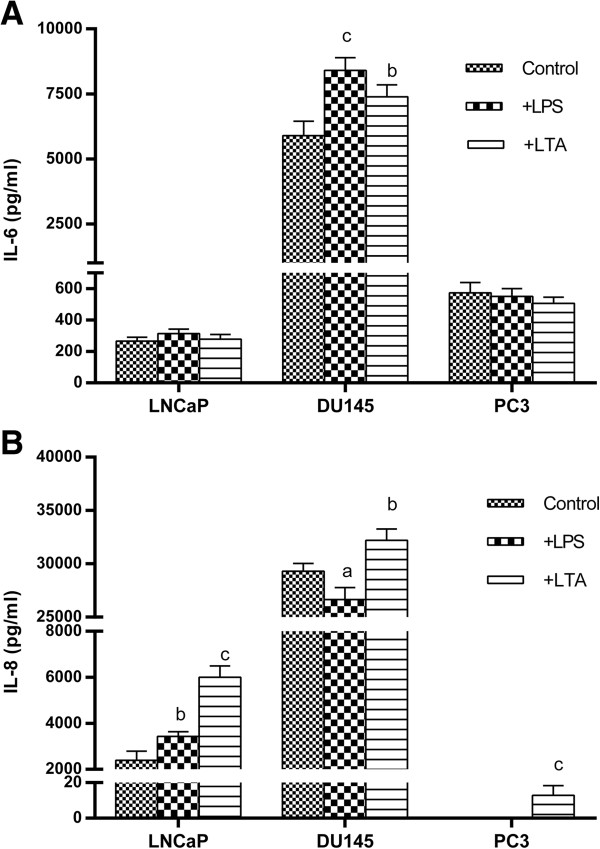
**Effects of TLR2 and TL4 ligation on cytokine production by human prostate cancer cell lines.** Prostate cancer cells were treated with LPS or LTA and concentrations of IL-6 **(A)** and IL-8 **(B)** were measured in culture supernatants by ELISA. Each bar represents the mean ± SD of three independent experiments and each test was done in triplicate. In each cell line, the data were compared to vehicle-treated (control) cells. c: p < 0.001, b: p < 0.01, a: p < 0.05.

Inflammatory cytokines such as IL-6, IL-8 and TNFα have major role in tumor biology and with this notion in mind; production of these cytokines by prostate cancer cell lines was studied at basal level and after stimulation with LPS and LTA. The results revealed that different prostate cell lines have different capacity to produce baseline levels of these cytokines and more interestingly respond in totally different manner to LPS or LTA stimulation. All cell lines failed to produce detectable levels of TNFα either in baseline levels or after stimulation with LPS or LTA. DU145 produced significantly higher basal levels of IL-6 compared to other cell lines (p < 0.001). Stimulation with LPS or LTA resulted in significantly higher levels of IL-6 production in DU145 cells (p < 0.001, p < 0.01), while such treatments had no considerable effect on IL-6 production by other two cell lines (Figure 4A). In a similar way to IL-6, highest amounts of IL-8 were produced by DU145 (p < 0.001). LPS treatment caused significant reduction of IL-8 production by DU145 (p < 0.05), while the opposite was true for LTA treatment (p < 0.01). LTA or LPS treatment caused significant increase in IL-8 production by LNCaP (p < 0.001, p < 0.01). PC3 failed to produce IL-8 at basal levels or after stimulation with LPS. LTA treatment, however, caused minimal detectable levels of IL-8 production by this cell line (Figure 4B).

### Effects of TLR2 and TL4 ligation on invasiveness of prostate cancer cells

**Figure 5:**
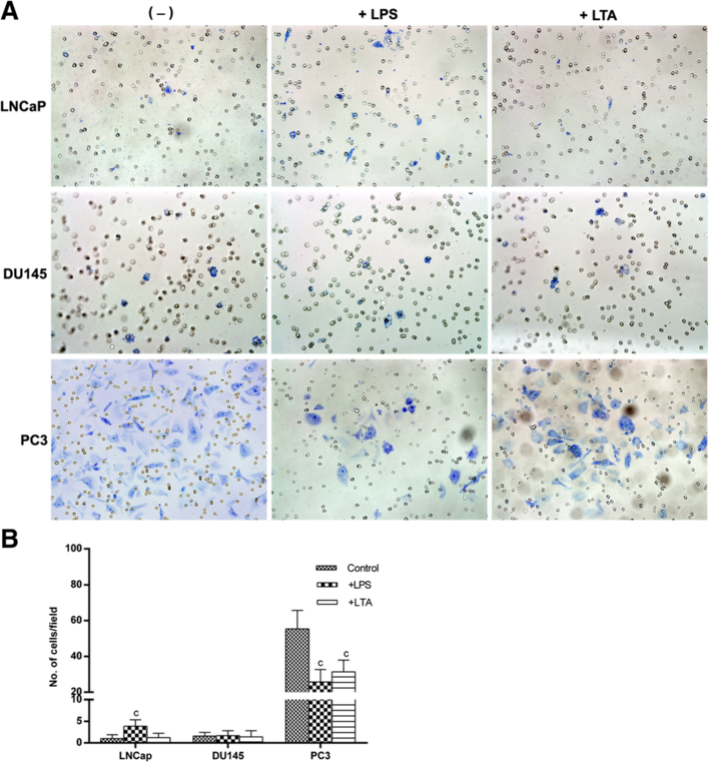
**Effects of TLR2 and TL4 ligation on invasive capacity of human prostate cancer cell lines. A**: Each cell line was treated with LPS or LTA during invasion assay. Invading cells were stained, photographed **(A)**, counted at 200 × in at least 50 fields and averaged **(B)**. Each bar represents the mean ± SD of three independent experiments. In each cell line, the data were compared to vehicle-treated (control) cells. c: p < 0.001.

Invasion is a process in which the cancer cells degrade extracellular matrix and invade to the surrounding tissue. Invasion is profoundly under the influence of microenvironment. In this regard Gram positive and negative bacteria may affect the invasive potential of tumors through stimulation of TLRs. With this notion, effect of LPS and LTA on invasive behavior of prostate cancer cell lines was investigated. The results of this experiment revealed that, basal invasion capacity of PC3 was significantly higher compared to other cell lines (p < 0.001) (Figure 5A). In PC3 cells, LPS treatment significantly reduced the capacity of the cells to invade (p < 0.001). The results of LTA stimulation was the same as LPS stimulation. LPS and LTA did not affect the invasion capacity of DU145. In LNCaP cells LPS but not LTA stimulation caused significant increase in invasion capacity (p < 0.001) (Figure 5B).

### Effects of TLR2 and TL4 ligation on prostate cancer cell adhesion

**Figure 6:**
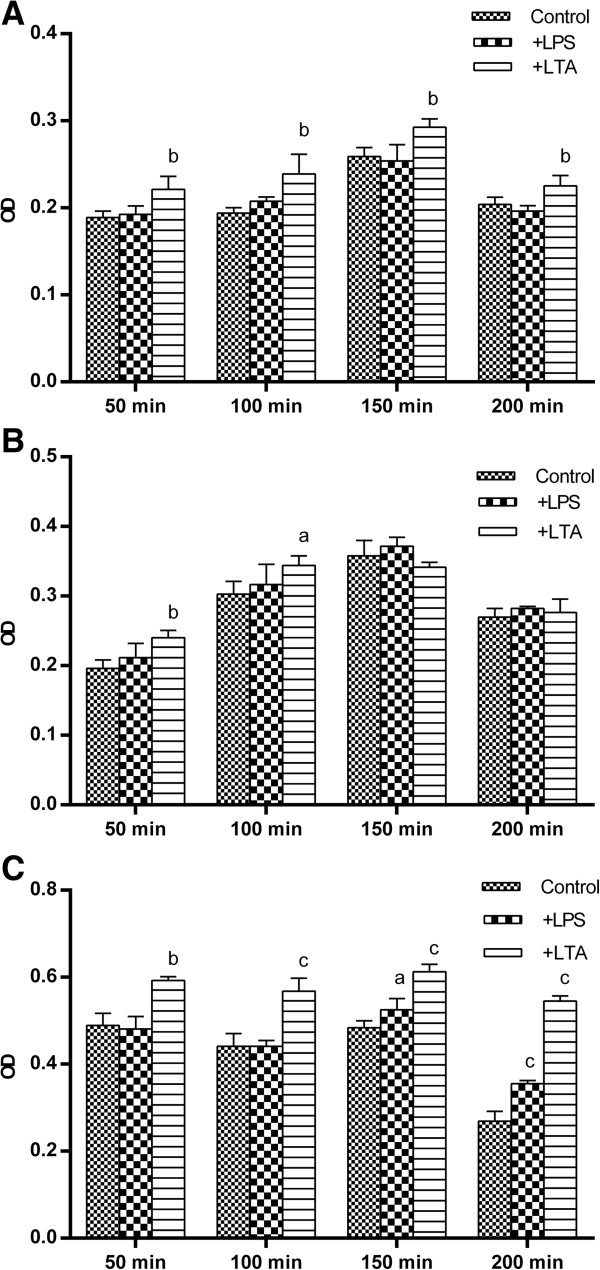
**Effects of TLR2 and TL4 ligation on adhesion capacity of human prostate cancer cell lines.** LNCaP **(A)**, DU145 **(B)** and PC3 **(C)** cells were treated with LPS or LTA and their adhesion capacity of cells was assesses in fibronectin-coated plates at different time intervals by colorimetric assay. Each bar represents the mean ± SD of three independent experiments and each test was done in triplicate. In each cell line, the data were compared to vehicle-treated (control) cells. c: p < 0.001, b: p < 0.01, a: p < 0.05.

In the next experiment, effect of LPS and LTA on adhesion of prostate cell lines to extra cellular matrix was surveyed. Different time points (50, 100, 150 and 200 min) was examined to find the optimal time point in which prostate cancer cell lines had the highest adhesion. All cell lines exhibited highest adhesiveness in 150 min, afterward cells gradually detached from the plate. In all time intervals, PC3 had the highest adhesive capacity compared to other cell lines. Stimulation of LNCaP with LTA but not LPS caused statistically significant increase in cell adhesion (p < 0.01) (Figure 6A). LTA also increased adhesive properties of DU145 (Figure 6B). Stimulation of PC3 with LTA and LPS caused statistically significant increase in cell adhesion (Figure 6C). To compare adhesive potential of different cell lines in response to LPS or LTA stimulation, the ratio of optical density of stimulated to non-stimulated state was calculated for each cell line and compared. The results of such comparison revealed that LTA has significantly higher positive effect on adhesive potential of PC3 compared to the other cell lines (p < 0.001) (data not shown).

## Discussion

Inflammation has long been known to be associated with the development of cancer. Multiple mechanisms are involved in inflammation-induced carcinogenesis; among others are anti-apoptotic effects of nuclear factor-κB (NF-κB), induction of oxidative damage to DNA and the induction of the tissue repair response [45]. Studies in human and animal models imply that inflammation might have a role in prostate cancer development and progression from organ-confined to metastatic disease [46-49]. Since prostate has proximity to the surfaces which could potentially be colonized by Gram negative and Gram positive bacteria, it is conceivable to hypothesize that infectious agents produce inflammation may be important factor in triggering prostate cancer. Whether infections, particularly ascending urethral infections, are responsible for prostate carcinogenesis remains an intriguing but unresolved question [50,51]. The potential role of genitourinary infection in the etiology of prostate cancer has been extensively investigated for 30 years. Despite the variable study designs and methodological approaches, there is no conclusive evidence for a direct link between infection and prostate cancer [52]. In most cases of prostatitis, no causal infectious agent can be identified making it difficult to link infection with prostate cancer in epidemiologic studies. However, an increased risk of prostate cancer in patients with sexually transmitted infections suggests that inflammation, rather than infection, initiates prostatic carcinogenesis [48,53,54]. In line with this possibility, intake of antioxidants or non-steroidal anti-inflammatory drugs has been associated with a decreased risk of prostate cancer [55,56].

Identifying the triggering events of inflammatory responses is one of the major challenges in understanding the connection between inflammation and cancer. Infection is the best characterized triggering factor of inflammation and TLRs are among the well-characterized receptors that initiate infection-mediated inflammatory responses [57]. Here we unrevealed some new aspects of functional consequences of TLR2 and 4 activation in LNCaP, DU145, and PC3 cells, which display low, moderate, and high metastatic potential, respectively [58]. We showed that different prostate cancer cell lines have different TLR expression profile, indicating their potential different capability for microenvironment sensing. Notably, TLR7 which sense synthetic compounds like Resiquimod (R-848) with potent anti-tumor activity was absent in all prostate cancer cell lines. Interestingly and consistent with the assumption that cancer cells may benefit from TLR signaling, the most aggressive prostate cell line, PC3, expressed the most comprehensive TLR sets. All cell lines expressed detectable levels of MyD88 implying that they might use MyD88-dependent TLR signaling pathways. Since in some cases there was no amplification in RT-PCR, possible polymorphisms in the annealing area of the primer pairs with no amplification in at least one cell line were checked in Ensemble gene browser (http://www.ensembl.org). Although there were some SNPs in the annealing area of the primers, such SNPs are unlikely to interfere with RT-PCR because: a) They were located in the middle part of primer binding sites and not in the 3’ region. Mismatches at the 3’ terminus of a primer-template duplex are more detrimental to PCR than internal mismatches. b) All primers we used, successfully amplified the gene of interest in at least two (and in most instances three) out of four cell types we investigated. c) The annealing area of the some genes which were amplified in all cell types (eg: TLR10) also showed internal SNPs. So polymorphisms are unlikely to affect the results of RT-PCR. In spite of this, the possibility that some cell lines express certain isoform of a given transcript that is not recognized by the primers used in this study could not be ruled out.

Although the expression profile of multiple TLRs in cancer cells from different histologically origin has been investigated [59], few data are available on functional expression of TLR2 and TLR4 in prostate cancer cells. Here we showed that LNCaP and PC3 cells, although harbour TLR2 and TLR4 proteins, do not express these receptors on their surface. Although TLR2 and TLR4 is usually expressed on the cell surface of immune cells, there are a number of reports on intracellular expression of these receptors in various cell types such as coronary artery endothelial cells [60], intestinal epithelial cells [61] and neuroblastoma cell line [27]. Interestingly, LPS-induced TLR4 signaling in intestinal epithelial cells occurs at the site of the Golgi apparatus and LPS-mediated cellular activation requires ligand internalization that occurs via a lipid raft dependent formation of clathrin-coated pits and intracellular transport to the Golgi compartment [61]. In the same way, TLR2 and its ligand, LTA, are internalized to Golgi apparatus and this process seems to be lipid raft-dependent [62]. Our results supported this set of data by showing that TLR2 and TLR4 were mainly localized to the peri-nuclear region, corresponding to Golgi apparatus. Interestingly, Takeyama et al. [63] reported surface expression of TLR2 in PC3 cells which is in contrary with what we reported here. They used flow cytometry for assessment of surface expression of TLR2, while in our system flow cytometry results were confirmed with immunofluorescent staining.

It has been reported that expression levels of TLRs, particularly TLR2 and TLR4, in immune and non-immune cells are modified after exposure to LPS, PMA and pro-inflammatory cytokines. Our results showed that surface expression of TLR2 and TLR4 in prostate cancer cells is not affected by such treatments suggesting that TLR2 and TLR4 expressions are differentially regulated in a cell-specific fashion. We showed that, in spite of the absence of surface expression of TLR2 and TLR4, prostate tumor cells are responsive to LPS and LTA implying that activation is mediated through ligand internalization. Such internalization processes of TLR ligands have been reported in epithelial cells [64] and may represent a natural regulatory step in the process of LPS or LTA-mediated activation of prostate epithelial cells to avoid overwhelming pro-inflammatory stimulation by the ascending infections.

For two reasons, expression of a given TLR on cancer cells could not be always considered as an advantageous natural selection process. First, expression of TLRs on cancer cells may not be necessarily associated with functional responses to their cognate ligands [65], and the second is the conflicting roles that have been attributed to TLR ligation in human cancers [26,32,66] including prostate carcinoma [63]. Due to chronic tissue damage with subsequent repair processes that could evolve in uncontrolled cell proliferation, infection-induced inflammatory responses can potentially enhance tumorigenesis. In parallel, tissue damage that usually occurred during cancer growth can elicit TLR-dependent generation of inflammatory products associated with recruitment of leukocytes with tumor promoting potentials [67]. On the contrary, engagement of TLRs with their ligands has been shown to induce apoptosis of tumor cells or to cause tumor regression indirectly by recruiting NK and cytotoxic T cells [68]. In this context, we evaluated functional consequences of TLR2 and TLR4-mediated activation in prostate cancer cells including proliferation, cell attachment, invasion and pro-inflammatory cytokine production.

We showed that different prostate cancer cell lines have different responsiveness to activation by LPS or LTA. While LPS induced significant increase of cell proliferation only in DU145 cells, LTA at all concentrations exhibited proliferative effect on all prostate cells tested. LPS also increased cell invasion in LNCaP, whereas both LPS and LTA reduced invasiveness of PC3, the most invasive prostate cancer cell line. The precise mechanism of this differential behavior of prostate cancer cells is not clear at the moment. The prostate cancer cells investigated here are distinct prostate cell lines, with differing origin (LNCaP from a lymph node; PC3 from bone; and DU145 from brain [69], invasiveness and androgen sensitivity. More importantly, they have unique expression pattern of genes, cell surface markers, transcriptional and signaling regulatory network and proteome and secretome which may be related to the different microenvironment they isolated [70-72]. For instance, LNCaP cells maintains various metabolic pathways, while, PC3 cells are characterized by their unique expression of cytoskeleton-related genes [71] implying that they might behave differentially in response to different stimuli. Equally important, LNCaP, DU145 and PC3 prostate carcinoma cell lines exhibit differential basal NF-κB activation levels. The highest constitutive NF-κB activation level is detected in DU145 cells [64], consistent with our results showing that this cell type produced significantly higher basal and LTA- or LPS-induced levels of pro-inflammatory cytokine, IL-6, compared to the other two cells. Surprisingly, the functional responsiveness to LPS in DU145 cells occurred in the absence of TLR4 transcript implying that compensatory pathways are responsible for capture and internalization of LPS in this cell line. It has been shown that TLR2 serves as primary receptor for Gram-positive bacteria and their cell wall components, whereas TLR4 as the primary LPS receptor. Subsequently, it was evident that human HEK293 cells co-transfected with TLR2, CD14 and myeloid differentiation protein 2 (MD-2), became highly responsive to all LPS preparations even at 0.1-1 ng/ml. Therefore, MD-2 enables very sensitive TLR2/CD14-mediated recognition of LPS [73]. Also, in an elegant work conducted by Yang et al., it was reported that TLR2 is activated by LPS in a response that is enhanced by CD14 [74]. We showed that neutralization of LPS by polymyxin B abrogated proliferative effect of LPS on DU145 cells to the baseline level indicating that LPS could actually increase proliferative capacity of cells lacking TLR4. Interestingly, omission of exogenous CD14 significantly abolished cell proliferation induced by LPS suggesting synergistic effect of CD14 and LPS on TLR4^-^ DU145 cells proliferation. Taken together, it seems that although TLR4 is a prototype receptor for LPS, its absence could be compensated by other relevant receptors of TLR family.

Prostate cancer cell lines also exhibited differential basal adhesiveness to solid matrix. Their adhesive capacity was also differed considerably in response to LPS and LTA treatments. The highly metastatic PC3 cell line attached more rapidly than all other cell types then entered a phase of spontaneous cell detachment about three hours after initiation of cell culture. Cytoskeletal architecture plays an important role in regulating cell adhesion and PC3 cells are characterized by their unique cytoskeleton-related genes compared to the other prostate cancer cell lines [71]. Importantly, increased adhesion capacity of PC3 cell line following LPS or LTA stimulation was accompanied by its reduced invasion potential. In line with this finding, it has been reported that there is a positive correlation between the invasion into extracellular matrix and lack of cell adhesion [75]. In contrast to PC3, LPS treatment in LNCaP cells had positive effect on cell adhesion implying that cells from the histologically same origin differentially regulate their adhesion molecules in response to PAMPs. Promotion of cell adhesion following LPS activation has also been reported in colorectal cancer cell lines [38].

From the data presented here some important points can be inferred: First, TLR activation in a particular cancer cell line could be associated with different and in some instances contrasting consequences. In this context, focusing on only one behavior of cancer cells following TLR stimulation, as done by some researchers, may be misleading and overall effects on different fundamental aspects of cancer cell behavior should be considered for correct interpretation. Second, cancer cells originated from the same histologically origin exhibit heterogeneous response to the same TLR ligand. Therefore, a thorough and comprehensive judgment on how and to what extent a particular cancer is affected by TLR agonist could not be inferred by studying an individual cell line. And finally, based on the information provided in the literature and the data presented here, different cancer types may positively or negatively regulated by TLR activation, a conclusion that is supported by extensive debate on the role of TLR in cancer [68].

## Materials and methods

### Cell lines and culture

Human prostate cell lines, PC3, DU145 and LNCaP were purchased from national cell bank of Iran. PBMC, MACS-purified monocytes or HL-60 cell line (national cell bank of Iran) were used as positive control of TLR expression, where appropriate. Cells were cultured in RPMI 1640 supplemented with 10% FBS (GIBCO, USA), penicillin and streptomycine at 37°C and 5% CO2. In order to avoid endotoxin contamination and maintenance of their original characteristics, cells were passaged with disposable plastic wares not more than 4 times before their cryopreservation.

### RNA Extraction and Complementary DNA (cDNA) Synthesis

Total RNA from cell homogenates was extracted by standard protocol. Briefly, chloroform was added to and mixed with RNA-Bee (Biosite, Sweden) solution containing sufficient number of cells, total RNA was collected from liquid supernatant and precipitated by two steps of alcohol precipitation. Extracted RNA was dissolved in 20–50 μL of sterile water. In each run of RNA extraction, quality of RNA was evaluated by gel electrophoresis. Ten microliters of total RNA from each cell line was heated to 65°C and immediately cooled on ice. A volume corresponding to 2 μg of RNA was added to the cDNA Mix including 5× buffer (Fermentase, Vilnius, Lithuania), 2 m MdNTP mix (Roche, Penzberg, Germany), 2 mM Random hexamer (Cybergene, Stockholm, Sweden), and 20 U⁄mL RT M-MuLV (Fermentase) in a final volume of 10 μL. The mixture was then incubated in 42°C for 60 min. The cDNA was then kept at -20°C.

### Reverse transcriptase polymerase chain reaction (RT-PCR)

PCR was carried out according to the protocol we published recently with some modifications [76]. In brief, 1 μL cDNA and 1 μL of each pair of primers (adapted from [77] except TLR4, MyD88, CD14 and β-actin which are designed in our laboratory) (Table 1), equivalent to the final concentrations of 0.4 pM for β-actin (as loading control) and 0.2 pM for TLR1-10, MyD88 and CD14 were added to 12.5 μl of ready to use master mix (Ampliqon, Denmark). After initial heating at 94°C for 3 minutes, PCR was performed 30 (for β-actin) or 35 cycles (for TLR1-10, MyD88 and CD14) with annealing temperatures listed in Table 1. For all PCR programs, denaturation and extension temperatures of 94°C and 72°C, respectively were applied for 30 seconds. Final extension for 7 minutes was performed for all amplifications. PCR systems devoid of template cDNA and no amplification controls (no reverse transcriptase added) were included as negative controls.

**Table 1:** Sequence, amplicon size and annealing temperatures of primers used in this study

	**Forward (5′–3′)**	**Reverse (5′–3′)**	**Product size (bp)**	**Annealing Temp(°C)**
**TLR1**	CTATACACCAAGTTGTCAGC	GTCTCCAACTCAGTAAGGTG	220	60
**TLR2**	GTACCTGTGGGGCTCATTGT	CTGCCCTTGCAGATACCATT	191	62
**TLR3**	GATCTGTCTCATAATGGCTTG	GACAGATTCCGAATGCTTGTG	305	60
**TLR4**	GAGCTTTAATCCCCTGAGGCA	GATTGGATAAGATTGTGAGCC	306	54.8
**TLR5**	CTAGCTCCTAATCCTGATG	CCATGTGAAGTCTTTGCTGC	438	59
**TLR6**	AGGTGCCTCCATTATCCTCA	GAATCCATTTGGGAAAGCAG	211	59
**TLR7**	CATGCTCTGCTCTCTTCAACC	CGATCACATGGTTCTTTGGA	201	59
**TLR8**	GCCAGCGAGTCTCACTGAACT	GCCAGGGCAGCCAACATA	558	61
**TLR9**	TTCCCTGTAGCTGCTGTCC	ACAGCCAGTTGCAGTTCACC	207	58
**TLR10**	GGCCAGAAACTGTGGTCAAT	CTGCATCCAGGGAGATCAGT	199	61
**CD14**	AGAGTTCACAAGTGTGAAGC	CCTTGACCGTGTCAGCATA	373	58
**MyD88**	GACCCAGCATTGAGGAGGATTG	GCTTCTGATGGGCACCTGGAGAG	435	60
**βactine**	GTGGGGCGCCCCAGGCACCA	CTCCTTAATGTCACGCACGATTTC	538	60

### Cell treatment

Each cell line was treated with pre-determined concentrations of LPS (Cat No: L4391 from Sigma, USA) and LTA (Cat No: L2515 from Sigma) in a specified time period. In cell lines with no expression of surface CD14, LPS treatments were accompanied by treatment with 1 ng/ml recombinant CD14 (R&D, USA). Concentration of LPS and LTA and treatment period varied depending on the experiment. Control wells were treated with vehicle and the same concentration of soluble CD14. In some experiments, CD14 was omitted to investigate the synergistic effect of this molecule on LPS-induced cell proliferation. To rule out the possibility of contamination of LPS with other TLRs ligands, LPS was neutralized using Polymyxin B (Sigma, USA) and the level of cell proliferation in DU145 cells was then quantified.

### Invasion assay

6-well matrigel invasion chambers (BD, USA) were rehydrated by warm DMEM in cell culture incubator, at 37°C, 5% CO2 for 2 h. After removing the medium, 2 ml of DMEM containing 5% FBS and 2 ml of cell suspension containing 1.25 × 10^5^ cells/ml in DMEM were added to the wells and upper inserts, respectively. Each cell line was treated with 100 ng/ml of LPS, 1 μg/ml of LTA or vehicle during the culture period. Non-invading cells were then removed from the upper surface of the membranes by scrubbing with cotton-tipped swabs two times followed by washing with warm culture medium. The cells on the lower surface of the membranes were fixed with ice cold methanol and stained with 1% toluidine blue each for 4 min followed by air drying. Membranes were removed from the insert housing by scalpel and mounted on the microscope slides. Invading cells were counted at 200 × in at least 50 fields and averaged.

### Adhesion assay

Effect of LPS and LTA on attachment capacity of prostate cancer cell lines was examined by cell attachment assay using fibronectin-coated 96-well attachment plates (BD). One day before assay, cells were passaged in logarithmic phase and after overnight culture were treated with 100 ng/ml LPS or 1 μg/ml LTA for 4 h. Cell suspensions were prepared and the cell count was adjusted to 2.5 × 10^5^/ml in DMEM. One hundred μl of cell suspension was added to each well in triplicate and plates were incubated with lid off at 37°C, 5% CO2 for 50, 100, 150 and 200 min. After gentle washing with warm PBS to remove non-adherent cells, adherent cells were fixed with 2% (v/v) formaldehyde for 10 min. After being washed three times with water, cells were stained with 0.1% crystal violet for 30 min. Wells were washed as above and dye was solubilized in 10% (v/v) acetic acid on orbital shaker at 150 rpm for 5 min. The extent of adhesion was evaluated by measuring the optical density at 595 nm.

### Cytokine assay

Cells suspended in RPMI containing 10% FBS were seeded in 96 well tissue culture plates at 50000 cell/well. After overnight culture, cells were treated with 100 ng/ml LPS or 1 μg/ml LTA for 48 h. Some cells remained untreated as control. Supernatants were collected, centrifuged and stored in -70°C for cytokine assay. Amounts of TNF-α, IL-8 (BD) and IL-6 (R&D, USA) in culture supernatant were measured according to the protocol provided by the manufacturer.

### SDS-PAGE and Western blotting

SDS gel electrophoresis and Western blotting were performed according to the protocol we published elsewhere [78]. Briefly, 2.0 ×10^7^ cells were homogenized in 1 ml complete RIPA Lysis Buffer System (Santa Cruz, USA) containing, 1.941 mM PMSF, 0.97 mM sodium orthovanadate and 1% (v/v) protease inhibitor cocktail and the protein content of cleared supernatants was determined by BCA protein assay (Pierce Biotechnology, USA). The samples were then separated on 10% SDS gel under reducing condition and transferred to the PVDF membrane. The membranes were blocked with 5% non-fat dry milk and probed with 1:100 dilution of goat anti-human TLR2 (Santa Cruz, USA) or 1 μg/mL goat anti-human TLR4 (R&D, USA) for 2 h. Membranes were consequently incubated with peroxidase-conjugated rabbit anti-goat secondary antibody (Avicenna research Institute, Iran) at a dilution of 1:10000. For protein loading control, membranes were stripped and re-probed with rabbit anti-human beta actin antibody (Sigma, USA) and peroxidase-conjugated sheep anti-rabbit Ig (Avicenna Research Institute) as above. The resulting signals were visualized using ECL Detection kit (GE Healthcare, UK) according to the manufacturer’s instruction. In negative control lanes, the primary antibodies were substituted by pre-immune goat serum.

### Flow cytometric analysis of TLR2 and TLR4 expression

In order to determine surface expression of TLR2 and TLR4, flow cytometric analyses were performed. All incubations were performed on ice. For TLR2 staining, the cells were incubated with Alexa fluor 488 conjugated anti-TLR2 antibody (BD) for 30 min. For TLR4 staining, a two-step staining protocol using biotin-conjugated anti-TLR4 (BD) and PE-Cy5.5 conjugated streptavidin (Invitrogen, USA) was utilized. In negative reagent control tubes, isotype matched irrelevant antibodies were used instead of the primaries. In some settings cells were treated with 50 ng/ml IL-1 and 100 ng/ml LPS or 1 μg/ml LTA for 24 h before flow cytometric analysis. Signals were analyzed by Partec flow cytometer (Partec, Germany). As positive control, monocyte gate of PBMC was analyzed in parallel.

### Immunofluorescent staining

Immunofluorescent staining of TLR2 and TLR4 was performed as described elsewhere [79]. Briefly, cells were cytospinned and fixed with either ice cold acetone (for TLR2) or natural buffered formalin (for TLR4). Primary antibodies [goat anti-TLR2 (Santa cruze), 1:100 for overnight or goat anti-TLR4 mouse (R&D), 10 μg/mL for 3 h] were added followed by 1:50 dilution of FITC-labeled rabbit anti-goat Ig (Avicenna Research Institute) for 60 min. Cells were then washed and counterstained with 4'-6-diamidino-2-phenylindole (DAPI) (BD). Fluorescent signals were visualized and photographed by a BX51 Olympus microscope equipped with DP71 CCD camera.

### Assessment of cell proliferation

Cell suspension in phenol red-free DMEM (Sigma, USA) supplemented with 10% FBS was plated in 96 well flat-bottom tissue culture plates (BD) at a pre-determined concentration of 10^4^ cell/well in a final volume of 100 μl. The optimal cell density and incubation time were determined for each cell line by titration and kinetic experiments where optimal conditions yielded the optical densities (OD) between 0.3-0.8 in the logarithmic portion of the standard curve. After that, 100 μl of phenol red-free DMEM containing LPS or LTA (final concentrations: 1–1000 ng/ml) was added to the test wells in triplicate, while control wells received the same volume of vehicle. After 48 hours, XTT was prepared at 1 mg/ml in pre-warmed (37°C) serum and phenol red-free DMEM. PMS was prepared at 5 mM (1.53 mg/ml) in PBS. Fresh XTT and PMS were mixed together at the appropriate concentrations. For a 0.025 mM PMS-XTT solution, 25 μl of the stock 5 mM PMS was added per 5 ml of XTT (1 mg/ml). 50 μl of this mixture (final concentration, 50 μg of XTT and 0.38 μg of PMS per well) was added to each well and the plates were incubated at 37°C, 5% CO2 for 4 hours. Thereafter, the OD values were read at 450 nm. For each cell line, the mean OD value of wells without cells was subtracted from that of test and control wells to calculate net OD value of each well.

### Statistical analysis

The SPSS version 13.0 (SPSS Inc., Chicago, IL) was used for all analyses. In all quantitative experiments the results represent data of three independent experiments and each test was done in triplicate. In case of invasion assay which was performed in three independent experiments but not as triplicate in each individual test, 50 fields were enumerated for each well. So, for statistical analysis, one-way analysis of variance (ANOVA) was used for group wise comparisons, followed by the appropriate post hoc tests (Dunnett’s). Figures are represented by Graphpad Prism Version 3 (GraphPad Software, SanDiego, CA). Results were considered significant at p < 0.05.

## Competing interests

The authors declare that they have no competing interests.

## Authors’ contributions

SR, NR, EM, OZ, JGh and LK performed the experiments and acquired data. SR prepared the first draft of manuscript. NA contributed to the study design. SZ contributed to the data analysis and interpretation. A-HZ conceived and designed the study, interpreted the data, critically reviewed and revised the manuscript and gave final approval. All authors read and approved the final manuscript.
